# Prognostic Nutritional Index and Postoperative Complications After Colorectal Surgery: A Retrospective Cohort Study

**DOI:** 10.3390/jcm15166430

**Published:** 2026-08-20

**Authors:** Joanna Braszczyńska-Sochacka, Aleksandra Goławska, Zofia Mik, Miłosz Lewandowski, Michał Mik

**Affiliations:** 1 Department of General and Colorectal Surgery, Medical University of Lodz, 90-549 Lodz, Poland; milosz.lewandowski@umed.lodz.pl (M.L.); michal.mik@umed.lodz.pl (M.M.); 2 Faculty of Health Sciences, Medical University of Lodz, 90-419 Lodz, Poland; 3 Faculty of Medicine, Medical University of Lodz, 90-419 Lodz, Poland

**Keywords:** prognostic nutritional index, colorectal cancer, postoperative complications, nutritional status, perioperative risk

## Abstract

**Background**: The prognostic nutritional index (PNI) is an inexpensive marker derived from serum albumin and peripheral lymphocyte count. Although low PNI has been associated with adverse surgical outcomes, its incremental value beyond basic clinical variables and the stability of commonly used thresholds remain uncertain in heterogeneous colorectal surgical populations. We evaluated the association between preoperative PNI and postoperative complications and examined whether adding PNI improved a basic clinical model. **Methods**: This retrospective single-center cohort included 205 consecutive adults undergoing colorectal surgery between January 2024 and March 2026. PNI was calculated as albumin (g/L) + 5 × lymphocyte count (10^9^/L). PNI was modeled primarily as a continuous predictor; PNI < 45 was examined secondarily. ROC analysis, multivariable logistic regression, nested-model comparison, calibration assessment, bootstrap internal validation, complete-case sensitivity analysis, and exploratory subgroup analyses were performed. **Results**: Fifty-six patients (27.3%) had PNI < 45. Postoperative complications occurred in 67 patients (32.7%) overall and were more frequent with PNI < 45 (51.8% vs. 25.5%; RR 2.03, 95% CI 1.40–2.95; OR 3.14, 95% CI 1.65–5.95; Fisher *p* < 0.001). PNI alone showed modest discrimination (AUC 0.659); the Youden cutoff was 45.45 (sensitivity 50.7%, specificity 78.3%). In the complete-case multivariable model (*n* = 191), each 5-point decrease in PNI was associated with higher odds of complications (OR 1.52, 95% CI 1.22–1.90; *p* < 0.001). Adding continuous PNI to age, BMI, and operative approach increased AUC from 0.663 to 0.711 and improved model fit (likelihood-ratio χ^2^ = 15.89, *p* < 0.001). Bootstrap resampling showed substantial cutoff variability (95% percentile interval 32.05–52.00). **Conclusions**: Lower preoperative PNI was associated with postoperative complications and added discriminatory information to a basic clinical model. However, its stand-alone discrimination was modest, the data-derived cutoff was unstable on bootstrap resampling, and residual confounding and clinical heterogeneity limit causal or treatment-directed interpretation. PNI should therefore be considered a risk marker rather than a stand-alone decision rule. Prospective interventional studies in well-defined patient groups are needed to determine whether PNI-guided nutritional optimization improves outcomes.

## 1. Introduction

The prognostic nutritional index (PNI) was introduced more than four decades ago as a simple measure combining serum albumin concentration and peripheral lymphocyte count [1]. Although it is often described as a nutritional index, both components are also influenced by inflammation and immune status, and PNI is increasingly framed as reflecting nutritional status, immune competence, and chronic inflammatory burden together rather than nutrition in isolation [2,3]. This is particularly relevant in colorectal surgery, where malignancy, bowel obstruction, infection, and chronic inflammatory disease may affect albumin and lymphocyte values before an operation. In clinical practice, PNI has the practical advantage of being calculated from routine laboratory tests and does not require additional investigations.

A substantial amount of literature has linked a low preoperative PNI with postoperative morbidity and long-term outcome across the gastrointestinal malignancy spectrum, including gastric, colorectal, breast, and lung cancers [1,2,3,4,5,6,7,8,9,10,11,12]. However, most of this evidence comes from studies that evaluated PNI within a single tumor type or a single surgical setting, which limits how confidently the reported associations generalize across different indications and procedures. The reported cutoff is also not uniform: in colorectal cancer studies it is frequently close to 45, whereas different thresholds have been used in patients with inflammatory bowel disease [13,14]. Part of this variation is likely to reflect differences between the populations being studied rather than a single biological boundary. A patient undergoing planned resection of localized colorectal cancer is clinically different from a patient operated on for obstruction or a patient with active inflammatory bowel disease. These differences are relevant when interpreting both PNI values and their relationship with postoperative risk.

Most available studies are observational, and this creates an important distinction between identifying risk and modifying it. An association between a low PNI and postoperative complications does not necessarily mean that PNI is sufficiently accurate to be used on its own for clinical decisions, nor does it show that nutritional treatment aimed at increasing PNI will reduce postoperative morbidity. Within colorectal surgery specifically, evidence on the predictive value of PNI for postoperative complications remains heterogeneous, and reported complication rates after colorectal procedures vary widely, with several large series placing overall morbidity in the region of 20–40% [15], underscoring a genuine opportunity to improve preoperative risk assessment and perioperative optimization. We therefore considered it important to examine not only the association between PNI and complications but also whether PNI contributes information beyond a small set of routinely available clinical variables.

We analyzed consecutive patients undergoing colorectal surgery for a range of malignant and non-malignant indications. Our primary aim was to assess whether preoperative PNI is associated with postoperative complications, with PNI treated primarily as a continuous variable, and whether incorporating PNI into a conventional clinical model based on age, BMI, and operative approach improves its discriminatory ability. We additionally examined the commonly used threshold of 45, assessed calibration and internal validity, and performed exploratory subgroup analyses when the available data allowed this. By evaluating the incremental value of PNI in this way, we aimed to inform risk-stratified perioperative assessment and to identify where targeted nutritional prehabilitation might plausibly be studied to reduce morbidity after colorectal surgery.

## 2. Materials and Methods

### 2.1. Study Design

This study was designed as a retrospective, single-center cohort analysis of consecutive adult patients who underwent colorectal surgery at the Department of General and Colorectal Surgery, Medical University of Lodz.

The study period extended from 1 January 2024 to 31 March 2026, spanning 27 months. All eligible patients treated within this timeframe were included in the analysis.

Data were collected retrospectively from electronic medical records and hospital databases.

According to local regulations, ethical approval and the requirement for informed consent were waived due to the retrospective design of the study and the use of anonymized data.

### 2.2. Eligibility and Cohort Assembly

During the 27-month study period, 218 patients underwent colorectal surgery at the study center; 13 patients (6.0%) were excluded because of missing preoperative serum albumin or lymphocyte values required for PNI calculation, leaving 205 patients for the final analysis.

Inclusion and exclusion criteria

- Inclusion: Patients who underwent their first colorectal surgical procedure at the study center during the specified period.
- Exclusion: Patients with missing preoperative data essential for PNI calculation (specifically, missing preoperative serum albumin or lymphocyte measurements) were excluded. Potential perioperative confounding factors, including ASA physical status, smoking status, neoadjuvant oncological treatment, and immunosuppressive therapy, were not considered in the current analysis due to incomplete availability of these data in the electronic medical records. Consequently, these variables could not be incorporated into the regression models.

### 2.3. Data Collection and Variables

Data on the following variables were retrospectively extracted from electronic medical records: age, sex, BMI, surgical indication (ICD-10), operative approach, documented preoperative weight loss, serum albumin, total protein, lymphocyte count, postoperative hospital stay, postoperative complications, and ICU admission.

Anthropometric assessment used BMI, categorized according to World Health Organization (WHO) criteria as underweight (<18.5 kg/m^2^), normal weight (18.5–24.9 kg/m^2^), overweight (25.0–29.9 kg/m^2^), and obesity (≥30.0 kg/m^2^); malnutrition was defined as BMI <18.5 kg/m^2^ [16].

Operative approach was classified as open, laparoscopic, or robotic. Procedures converted from laparoscopy to laparotomy were analyzed with the open group for multivariable modeling, because the final operative exposure was open surgery.

Laboratory parameters—serum albumin, total protein, and lymphocyte count—were collected one day prior to surgery.

The primary outcome was any postoperative complication coded as present in the source dataset, encompassing surgical site infection (superficial or deep wound infection), wound dehiscence (evisceration), anastomotic leakage, intra-abdominal abscess, enteric fistula, bleeding from the anastomotic site, postoperative bowel dysfunction, and reoperation. Formal Clavien–Dindo grades were not prospectively recorded. Because the available free-text documentation was not sufficiently standardized to assign severity grades reproducibly without retrospective clinical inference, complications were not reclassified into Clavien–Dindo categories in the revised analysis, which limits direct comparison with severity-graded studies in the literature.

Malignant versus non-malignant disease was defined from the recorded ICD-10 diagnosis (codes beginning with C were classified as malignant). Emergency/elective status was analyzed only when explicitly available in the source dataset; this variable was missing in 82 of 205 patients, and subgroup results by urgency are therefore exploratory and should not be generalized to the entire cohort.

Length of postoperative hospital stay was also recorded.

BMI was missing for 14 patients (6.8%), because preoperative height was not documented. These patients were excluded on a pairwise basis from analyses involving BMI but were retained for all other analyses. No imputation was performed.

### 2.4. Prognostic Nutritional Index

PNI was calculated as serum albumin (g/L) + 5 × lymphocyte count (10^9^/L), consistent with commonly used unit conventions [10,11]. PNI was modeled primarily as a continuous predictor to avoid information loss from dichotomization. For clinical interpretability and comparison with prior literature, PNI < 45 was evaluated as a secondary analysis.

### 2.5. PNI Cutoff and Modeling

For the secondary threshold analysis, the data-derived PNI cutoff was identified from the ROC curve using the maximum Youden index. Because deriving and evaluating a threshold in the same cohort may overstate its stability, the cutoff was internally assessed by 2000 bootstrap resamples. The prespecified threshold of PNI < 45 was retained as the clinically interpretable secondary threshold.

Discrimination was quantified using the area under the ROC curve (AUC). Sensitivity and specificity were reported for the Youden-derived cutoff. Bootstrap percentile intervals were used to describe the stability of the data-derived cutoff and AUC.

### 2.6. Statistical Analyses

Continuous variables were summarized as means ± standard deviation or medians with interquartile ranges/ranges, as appropriate; categorical variables were summarized as counts and percentages.

Comparisons between groups were performed using the χ^2^ test or Fisher’s exact test for categorical variables, and the Mann–Whitney U test for continuous variables.

The association between the prognostic nutritional index (PNI) and clinical variables was assessed using Spearman’s rank correlation coefficient (ρ).

Relative risk (RR) and odds ratio (OR) with 95% confidence intervals (CI) were calculated to evaluate the association between low PNI and postoperative complications. Effect size for categorical associations was estimated using the phi (φ) coefficient.

Receiver operating characteristic (ROC) curve analysis was performed to assess the discriminative ability of PNI for predicting postoperative complications. The optimal cutoff value was determined using the Youden index (sensitivity + specificity − 1). The area under the ROC curve (AUC) with 95% CI was reported, along with sensitivity and specificity.

The primary multivariable logistic regression modeled PNI continuously together with age, BMI, and operative approach. The PNI coefficient is reported per 5-point decrease. A secondary model used PNI < 45. Predictive performance of the basic clinical model (age, BMI, operative approach) was compared with the same model plus continuous PNI using AUC and a likelihood-ratio test. Calibration was assessed with the Brier score and Hosmer–Lemeshow test. With 67 outcome events and four modeled predictors, the approximate events-per-variable ratio was 16.8. Because BMI was missing in 14 patients, the principal model used complete cases (*n* = 191); a sensitivity model excluding BMI was fitted in all 205 patients. Exploratory subgroup analyses examined malignant versus non-malignant disease, open versus minimally invasive surgery, and elective versus emergency surgery when urgency data were available.

A *p*-value < 0.05 was considered statistically significant.

## 3. Results

### 3.1. Study Cohort and Baseline Results

A total of 205 patients were included in the study over a 27-month period (January 2024–March 2026), of whom 101 (49.3%) were female and 104 (50.7%) were male. The mean age was 63 ± 16 years, with a median of 67 years (range 20–91). The mean length of hospital stay was 11 ± 13 days (median 7 days, range 3–140).

Using the prespecified secondary threshold, 56 patients (27.3%) had PNI < 45 and 149 (72.7%) had PNI ≥ 45. Postoperative complications occurred in 67 patients (32.7%) overall, including 29/56 (51.8%) with PNI < 45 and 38/149 (25.5%) with PNI ≥ 45.

Regarding body mass index, 9 patients (4.4%) were underweight, 77 (37.6%) had normal weight, and 105 (51.2%) were overweight or obese; BMI data were unavailable for 14 patients (6.8%).

The most common surgical approach was laparotomy (*n* = 91, 44.4%), followed by laparoscopy (*n* = 77, 37.6%) and robotic surgery (*n* = 34, 16.6%). Conversion from laparoscopy to open surgery was required in three patients (1.5%). Complication rates were highest in the laparotomy group (45.1%), followed by laparoscopic procedures (23.4%) and robotic (17.6%). Baseline characteristics are summarized in Table 1.

### 3.2. Association Between PNI and Postoperative Complications

A total of 205 patients with complete data on PNI and postoperative complications were included in the analysis.

Postoperative complications occurred in 67 patients.

Patients with PNI < 45 had a significantly higher complication rate than those with PNI ≥ 45 (51.8% vs. 25.5%).

Low PNI was associated with approximately twice the risk of complications (RR 2.03; 95% CI 1.40–2.95) and higher odds of complications (OR 3.14; 95% CI 1.65–5.95).

The association remained statistically significant by Fisher’s exact test (*p* < 0.001).

### 3.3. ROC Curve Analysis and Optimal PNI Cutoff

**Figure 1:**
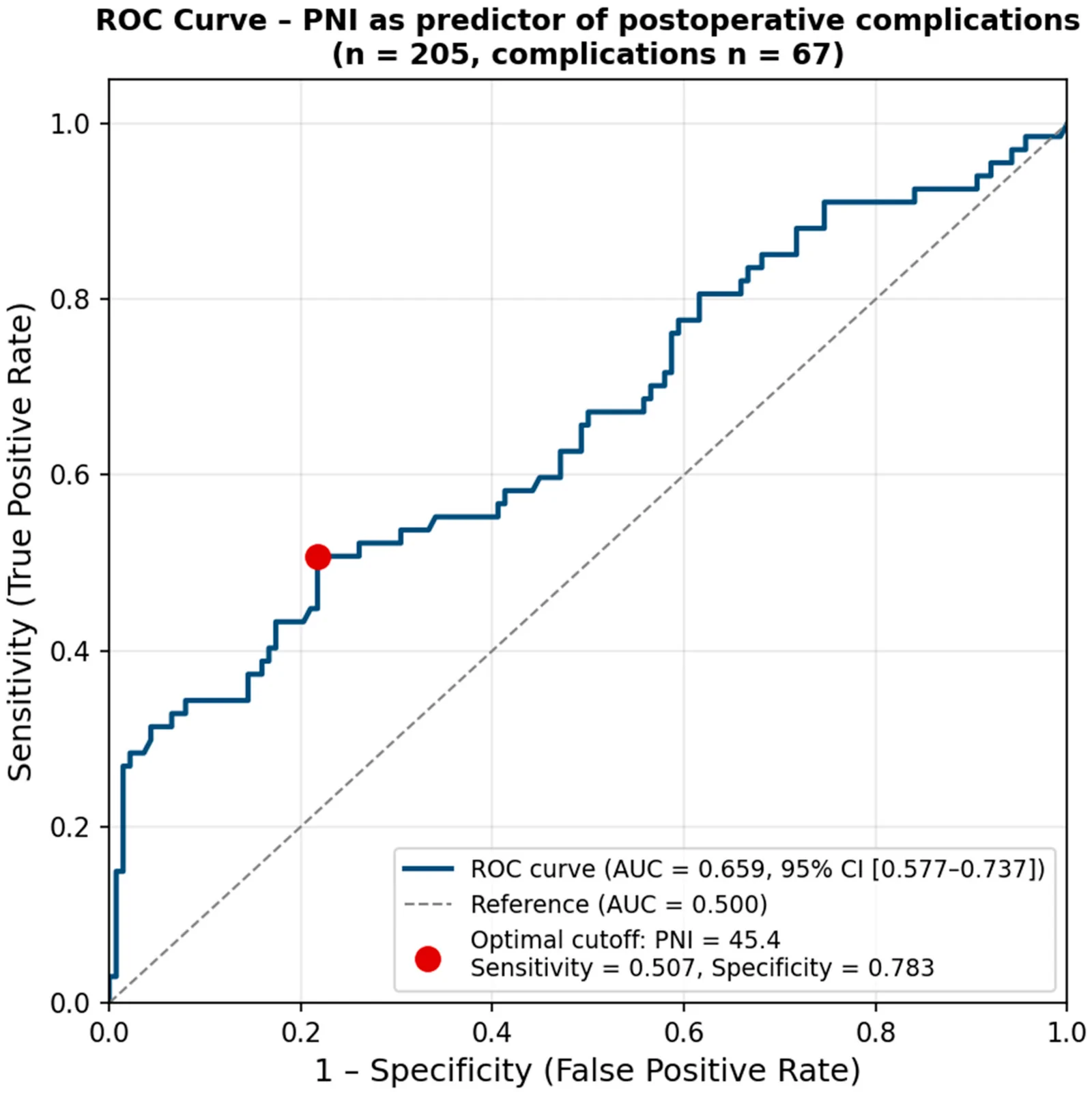
ROC curve for PNI predicting postoperative complications.

PNI alone showed modest discrimination for postoperative complications (AUC 0.659) (Figure 1).

The maximum Youden index identified a cutoff of PNI 45.45, with sensitivity 50.7% and specificity 78.3%. Across 2000 bootstrap resamples, the median selected cutoff was 45.45 and the 95% percentile interval was 32.05–52.00; the bootstrap AUC 95% percentile interval was 0.574–0.741. This wide cutoff interval indicates that 45.45 should not be interpreted as a stable population-specific decision threshold.

### 3.4. Univariable Analyses

Lower PNI values were significantly associated with adverse clinical outcomes.

Patients requiring intensive care unit (ICU) stay had significantly lower PNI values compared to those who did not (median: 37.25 vs. 49.55; *p* = 0.00070).

PNI was moderately negatively correlated with length of hospital stay (Spearman ρ = −0.422, *p* < 0.000001), indicating that lower PNI was associated with longer hospitalization.

A weak but statistically significant association was observed between PNI and type of surgical procedure (*p* = 0.0218).

PNI showed a weak-to-moderate positive correlation with BMI (ρ = 0.343, *p* = 0.0000012) and a weak negative correlation with age (ρ = −0.239, *p* = 0.00057).

The results of the univariable analyses are presented in Table 2.

### 3.5. Multivariable Analysis

In the primary complete-case multivariable model (*n* = 191), lower PNI remained associated with postoperative complications after adjustment for age, BMI, and operative approach. Each 5-point decrease in PNI was associated with 1.52-fold higher odds of complications (95% CI 1.22–1.90; *p* < 0.001). Open surgery was also associated with higher odds of complications (OR 2.53; *p* = 0.006), whereas age and BMI were not statistically significant. In the secondary dichotomous model, PNI <45 was associated with complications (adjusted OR 2.75; 95% CI 1.33–5.70; *p* = 0.006). The results of the multivariable analyses are presented in Table 3.

### 3.6. Incremental Value, Calibration, and Sensitivity Analyses

In the 191 patients with complete BMI data, the basic model containing age, BMI, and operative approach had an AUC of 0.663. Adding continuous PNI increased the AUC to 0.711. The likelihood-ratio test favored the PNI-augmented model (χ^2^ = 15.89, 1 df, *p* < 0.001). Model calibration was acceptable by the Hosmer–Lemeshow test (χ^2^ = 8.79, 8 df, *p* = 0.360), and the Brier score was 0.186. In a sensitivity model fitted to all 205 patients after omitting BMI, each 5-point decrease in PNI remained associated with complications (OR 1.36, 95% CI 1.14–1.63; *p* < 0.001).

### 3.7. Exploratory Subgroup Analyses

The association between PNI < 45 and postoperative complications was generally in the same direction across the major clinical subgroups, although the estimates were imprecise and confidence intervals were wide. The crude OR was 1.63 (95% CI 0.67–3.95) in malignant disease and 6.14 (95% CI 2.15–17.52) in non-malignant disease; 2.76 (95% CI 1.18–6.46) after open surgery and 2.08 (95% CI 0.69–6.30) after minimally invasive surgery; and 3.67 (95% CI 1.50–8.99) among elective procedures. Only 14 emergency procedures had documented urgency, including no complications among the single PNI ≥ 45 patient, preventing a stable OR estimate. Urgency was missing in 82/205 patients; these subgroup analyses are therefore exploratory and were not used to infer effect modification (Table 4).

## 4. Discussion

In this cohort of 205 colorectal surgical patients, lower preoperative PNI was associated with postoperative complications. Treating PNI continuously, rather than relying only on a threshold, preserved information and showed that each 5-point decrease was associated with approximately 52% higher adjusted odds of complications. Association and prediction should nevertheless be distinguished: PNI alone had only modest discrimination (AUC 0.659), while its addition to a basic clinical model improved AUC from 0.663 to 0.711 and significantly improved nested-model fit.

Our findings are in keeping with previous reports from colorectal surgery. Tokunaga et al. [6] and Cao et al. [7] found that lower PNI was associated with adverse outcomes after colorectal cancer surgery. Similar conclusions have emerged from broader analyses by Zhao et al. [8] and Sun et al. [3], and prognostic associations have also been reported by Kazi et al. [9] and Mohri et al. [11]. Given the amount of existing evidence, the present study should not be viewed simply as another confirmation that low PNI identifies higher-risk patients. Rather, we sought to determine how the marker behaved in a mixed colorectal surgical population and whether it added useful information to readily available clinical variables.

The cutoff of 45 should not be regarded as a fixed biological threshold. Published values differ between studies, with cutoffs around 45–47 in some colorectal cancer series and lower values in inflammatory bowel disease [13,14]. Differences in diagnosis, inflammatory activity, cancer burden, obstruction, urgency of surgery, and baseline nutritional status can all shift the distribution of PNI within a cohort. The method used to select a cutoff is another source of variation. In our data, the bootstrap distribution of the Youden-derived threshold was wide. We therefore retained 45 mainly because it is clinically familiar and facilitates comparison with earlier work, rather than because our data establish it as the optimal threshold for all colorectal patients.

Interpretation is also complicated by the mixed clinical profile of the cohort. Elective minimally invasive resection for colorectal cancer, surgery for benign disease, and an emergency operation for obstruction do not carry the same baseline risk. Malignant and non-malignant diagnoses could be separated in our dataset, but information on emergency versus elective surgery was unavailable in 82 patients. For that reason, the subgroup analyses are best treated as hypothesis-generating rather than confirmatory. They do not establish that PNI has the same prognostic meaning across these clinical settings, and larger studies with prospectively defined groups would be needed to address that question.

Using 45 as a descriptive threshold, 56 of 205 patients (27.3%) would have been classified as having a low PNI. This gives an estimate of how often a low PNI would be encountered among patients treated in everyday colorectal practice, but it does not equal the proportion of patients who actually require nutritional treatment. PNI does not replace a formal nutritional assessment, which should also consider weight loss, food intake, disease severity, and established screening instruments. Our retrospective data also cannot determine whether nutritional support given to patients with a low PNI would reduce complications. That question requires an interventional study.

The moderate AUC indicates that PNI should not be used as a stand-alone decision tool on the basis of these data. A more plausible role is as one component of a broader perioperative risk assessment. Although the model containing PNI performed better than the basic clinical model, internal validation indicated some optimism in the apparent discrimination. External validation in an independent cohort would therefore be required before the model could be considered for clinical prediction.

### 4.1. Strengths and Limitations

Several aspects of the analysis strengthen the study. Patients were included consecutively, both components required to calculate PNI are routinely measured, and PNI was examined both as a continuous variable and using a clinically familiar cutoff. We also compared models with and without PNI, assessed calibration, performed internal validation by bootstrap resampling, and conducted a sensitivity analysis addressing missing BMI data.

The results nevertheless need to be interpreted in light of several limitations. First, the retrospective design permits residual confounding. ASA class, smoking status, comorbidity burden, neoadjuvant treatment, immunosuppressive therapy, operative time, blood loss, and complete tumor stage information were not consistently available for adjustment. Accordingly, the observed association between PNI and postoperative complications reflects adjustment only for the covariates available in this dataset and should not be interpreted as evidence of independence from all clinically relevant confounders. Second, BMI was missing in 14 patients (6.8%) and no multiple imputation was performed. Third, emergency/elective status was missing in 82 patients, limiting subgroup inference. Fourth, the cohort pooled malignant and benign disease and different operative settings. Fifth, postoperative complications were recorded as a binary source variable with heterogeneous free-text descriptions; formal Clavien–Dindo grading was unavailable and could not be assigned reproducibly without retrospective inference. Sixth, the ROC-derived threshold was developed in the same cohort and showed wide bootstrap variability. Finally, internal bootstrap validation does not replace external validation.

### 4.2. Future Research and Clinical Application

Additional retrospective demonstrations that low PNI is associated with postoperative complications are unlikely, by themselves, to establish clinical utility. Future research should therefore move beyond retrospective confirmation of this association. Prospective studies in clearly defined patient groups, including interventional trials, are needed to determine whether nutritional support or prehabilitation targeted to patients with a low PNI can actually modify postoperative outcomes. Studies should also determine whether PNI adds value beyond validated perioperative risk scores and formal nutritional screening instruments, and whether clinically meaningful thresholds differ between elective cancer surgery, benign disease, inflammatory conditions, and emergency colorectal surgery.

## 5. Conclusions

Lower preoperative PNI was associated with a higher risk of recorded postoperative complications after colorectal surgery. In the available complete-case model, a 5-point decrease in PNI remained associated with complications after adjustment for age, BMI, and operative approach, and addition of PNI improved discrimination relative to the basic clinical model. Nevertheless, PNI alone showed only moderate discrimination, the data-derived cutoff was unstable on bootstrap resampling, and substantial residual confounding and cohort heterogeneity remain. PNI should therefore be regarded as a readily available risk marker that may complement, rather than replace, broader perioperative assessment. The present study does not demonstrate that PNI-guided nutritional intervention improves outcomes; this requires prospective interventional evaluation.

## Figures and Tables

**Table 1:** Baseline demographic, clinical, and perioperative characteristics of the study cohort according to preoperative Prognostic Nutritional Index category.

Characteristic	All Patients	PNI < 45	PNI ≥ 45
Total patients, *n*	205	56	149
Study period	1 January 2024–31 March 2026 (27 months)	—	—
Female	101 (49.3)	31 (55.4)	70 (47.0)
Male	104 (50.7)	25 (44.6)	79 (53.0)
Mean ± SD	63.0 ± 15.8	65.1 ± 15.7	62.2 ± 15.8
Median (range)	67 (20–91)	68.5 (32–90)	66 (20–91)
Mean ± SD	10.9 ± 12.5	17.1 ± 19.9	8.5 ± 6.9
Median (range)	7 (3–140)	12 (3–140)	6 (4–59)
With complications	67 (32.7)	29 (51.8)	38 (25.5)
Without complications	138 (67.3)	27 (48.2)	111 (74.5)
Missing data	14 (6.8)	9 (16.1)	5 (3.4)
Underweight (<18.5 kg/m^2^)	9 (4.4)	7 (12.5)	2 (1.3)
Normal weight (18.5–24.9 kg/m^2^)	77 (37.6)	24 (42.9)	53 (35.6)
Overweight/obese (≥25.0 kg/m^2^)	105 (51.2)	16 (28.6)	89 (59.7)
Laparotomy	91 (44.4)	38 (67.9)	53 (35.6)
With complications	41 (45.1)	23/38 (60.5)	18/53 (34.0)
Laparoscopy	77 (37.6)	13 (23.2)	64 (43.0)
With complications	18 (23.4)	4/13 (30.8)	14/64 (21.9)
Robotic	34 (16.6)	5 (8.9)	29 (19.5)
With complications	6 (17.6)	2/5 (40.0)	4/29 (13.8)
Conversion (laparoscopy→open)	3 (1.5)	0 (0.0)	3 (2.0)
2/3 (66.7)			

**Table 2:** Univariable analysis—association of PNI with clinical outcomes.

Variable/Clinical Outcome	Statistic	Value	*p*
ICU admission (yes vs. no)	Median PNI	37.25 vs. 49.55	0.0007
Length of hospital stay	Spearman ρ	−0.422	<0.000001
Type of surgical procedure	Group comparison	—	0.0218
BMI	Spearman ρ	+0.343	0.0000012
Age	Spearman ρ	−0.239	0.00057

ρ—Spearman’s correlation coefficient; ICU—intensive care unit.

**Table 3:** Multivariable analysis—logistic regression (outcome: postoperative complications).

Variable	Adjusted OR	*p*	95% CI
PNI (per 5-point decrease)	1.52	<0.001	1.22–1.90
Age (per year)	1.00	0.856	0.98–1.02
BMI (per kg/m^2^)	1.05	0.178	0.98–1.12
Open vs. minimally invasive surgery	2.53	0.006	1.30–4.91

OR—odds ratio; 95% CI—95% confidence interval; *p* < 0.05 considered statistically significant.

**Table 4:** Exploratory subgroup analyses of the association between PNI < 45 and postoperative complications. * Urgency was unavailable for 82 patients; urgency-stratified analyses are exploratory. OR, odds ratio; CI, confidence interval; PNI, Prognostic Nutritional Index.

Subgroup	*n*	PNI < 45: Complications/Non-Complications	PNI ≥ 45: Complications/Non-Complications	Crude OR (95% CI)
Malignant disease	134	10/18	27/79	1.63 (0.67–3.95)
Non-malignant disease	71	19/9	11/32	6.14 (2.15–17.52)
Open surgery	94	23/15	20/36	2.76 (1.18–6.46)
Minimally invasive surgery	111	6/12	18/75	2.08 (0.69–6.30)
Elective surgery *	109	17/11	24/57	3.67 (1.50–8.99)
Emergency surgery *	14	8/5	0/1	Not estimable

## Data Availability

The data presented in this study are available on request from the corresponding author.

## Abbreviations

The following abbreviations are used in this manuscript:

PNI	Prognostic nutritional index
BMI	Body mass index
WHO	World Health Organization
ALB	Serum albumin
TP	Total protein
LYMPH	Lymphocyte count
SSI	Surgical site infection
ROC	Receiver operating characteristic
AUC	Area under the ROC curve
PPV	Predictive value
NPV	Negative predictive value
IQR	Interquartile ranges
RR	Relative risk
OR	Odds ratio
CI	Confidence intervals
